# Experimental Challenge of Sheep and Cattle with Dugbe Orthonairovirus, a Neglected African Arbovirus Distantly Related to CCHFV

**DOI:** 10.3390/v13030372

**Published:** 2021-02-26

**Authors:** Julia Hartlaub, Felicitas von Arnim, Christine Fast, Ali Mirazimi, Markus Keller, Martin H. Groschup

**Affiliations:** 1 Institute of Novel and Emerging Infectious Diseases, Friedrich-Loeffler-Institut, Suedufer 10, 17489 Greifswald-Insel Riems, Germany; julia.hartlaub@fli.de (J.H.); felicitas.var@mail.de (F.v.A.); christine.fast@fli.de (C.F.); markus.keller@fli.de (M.K.); 2 Department of Medicine, Karolinska Institutet, SE-17177 Stockholm, Sweden; ali.mirazimi@ki.se; 3 National veterinary Institute, SE-75189 Uppsala, Sweden

**Keywords:** Dugbe orthonairovirus, DUGV, Crimean-Congo hemorrhagic fever orthonairovirus, CCHFV, cross-reactivity, animal model

## Abstract

Dugbe orthonairovirus (DUGV) is a tick-borne arbovirus within the order *Bunyavirales*. DUGV was first isolated in Nigeria, but virus isolations in ten further African countries indicate that DUGV is widespread throughout Africa. Humans can suffer from a mild febrile illness, hence, DUGV is classified as a biosafety level (BSL) 3 agent. In contrast, no disease has been described in animals, albeit serological evidence exists that ruminants are common hosts and may play an important role in the transmission cycle of this neglected arbovirus. In this study, young sheep and calves were experimentally inoculated with DUGV in order to determine their susceptibility and to study the course of infection. Moreover, potential antibody cross-reactivities in currently available diagnostic assays for Crimean-Congo hemorrhagic fever orthonairovirus (CCHFV) were assessed as DUGV is distantly related to CCHFV. Following subcutaneous inoculation, none of the animals developed clinical signs or viremia. However, all ruminants seroconverted, as demonstrated by two DUGV neutralization test formats (micro-virus neutralization test (mVNT), plaque reduction (PRNT)), by indirect immunofluorescence assays and in bovines by a newly developed DUGV recombinant N protein ELISA. Sera did not react in commercial CCHFV ELISAs, whereas cross-reactivities were observed by immunofluorescence and immunoblot assays.

## 1. Introduction

Dugbe orthonairovirus (DUGV) is a tick-borne arbovirus, which belongs to the genus *Orthonairovirus* within the order *Bunyavirales.* This genus includes viruses of veterinary importance (e.g., Nairobi sheep disease orthonairovirus), as well as members with public health impact, which are associated with severe human disease (e.g., Crimean-Congo hemorrhagic fever orthonairovirus) [1].

The genome of DUGV is composed of a tripartite single-stranded RNA. The segments, designated small (S), medium (M) and large (L), encode the nucleocapsid protein (N), envelope glycoproteins (Gn, Gc) and the RNA-dependent RNA polymerase, respectively [2,3].

DUGV, Kupe orthonairovirus (KUPV) and Nairobi sheep disease orthonairovirus (NSDV) belong to the NSDV serogroup. However, a distant serological relationship to the Crimean-Congo hemorrhagic fever orthonairovirus (CCHFV) serogroup, which includes Crimean-Congo hemorrhagic fever orthonairovirus (CCHFV) and Hazara orthonairovirus (HAZV), is presumed [4,5,6].

In 1964, the prototype strain of DUGV (IB AR 1792) was isolated from a pool of *Amblyomma variegatum* ticks sampled at the cattle market in Ibadan, Nigeria [7]. Indeed, DUGV is considered to be the most often isolated arbovirus in Nigeria [8]. DUGV has additionally been isolated in ten other African countries; namely Kenya, Central African Republic, Ethiopia, Uganda, Ghana, Guinea, Senegal, Sudan, Cameroon and Chad [9,10,11,12,13,14,15,16,17]. Serological data indicate that DUGV is also present in Egypt and South Africa, suggesting that this virus circulates widely throughout the African continent [18,19].

Whilst most of the isolates were obtained from ticks collected from ruminants, DUGV could only be occasionally isolated from animals (cattle [7], wild rodents [11,20], a monkey [16] and a bird [11]), implying that this virus can infect a fairly wide host range. DUGV has also been isolated from other arthropods (*Culicoides* and *Culicidae*) [7], but vector competence studies revealed that *Aedes aegypti* mosquitoes were refractory to DUGV infection [21]. These insects might be mechanical carriers of the virus after blood-feeding from viremic animals, but do not seem to play a role in the natural transmission cycle.

Due to its zoonotic potential, DUGV is classified as a biosafety level (BSL) 3 agent. Several laboratory-acquired infections as well as field infections associated with febrile illness are documented [7,18,22,23]. In South Africa, one case of a patient suffering from high fever, encephalitis and prolonged thrombocytopenia was investigated. Even if DUGV could not be successfully isolated, a monospecific rise of anti-DUGV antibodies without detectable antibodies against CCHFV supports that the disease was indeed caused by DUGV [18]. As DUGV seems to occur widely in Africa, the virus might actually be the causative agent of more cases of fever of unknown origin (FUO).

The majority of the studies reporting virus isolations or serological investigations were conducted between the 1970s and early 1990s; hence, DUGV is quite a neglected virus nowadays. To determine the distribution of DUGV, RT-qPCR screening of ticks as well as the serological monitoring of animals (e.g., ruminants) can give a hint as to where human infections might occur. To date, mainly complement fixation (CF) and hemagglutination inhibition (HI) have been employed for the detection of DUGV antibodies, highlighting the need for the development of new sensitive and specific diagnostic assays.

As DUGV is distantly related to CCHFV (59% and 70% amino acid identity for the complete sequence of the S segment and partial genome sequences of the L segment, respectively) [24,25], arouses concerns that antibodies directed against DUGV might interfere with current CCHFV diagnostics and lead to false-positive CCHFV serological diagnoses. To monitor the current CCHFV distribution and thereby evaluate the risk of human infections with this highly pathogenic virus (BSL 4), serological studies involving ruminants are being conducted worldwide [26]. As CCHFV is prevalent in many African countries, there is an overlap in the distribution range of DUGV and CCHFV. Hence, it is important to investigate putative cross-reactivities.

Former studies have led to quite inconsistent results. Two studies conducted in South Africa and the Central African Republic (CAR) used ELISA systems based on acetone- or β-propiolactone-treated virus particle antigens to detect DUGV and CCHFV antibodies in cattle. In South Africa, the majority of the animals (93%), which tested positive for CCHFV antibodies did not have anti-DUGV antibodies—indicating that cross-reactivities do not play a major role [18]. In contrast, the study from the CAR revealed that 96% of cattle sera (>2 years) with anti-CCHFV antibodies also reacted with the DUGV antigen. The authors of this study concluded that this seems to occur due to cross-reactivities rather than due to co-infections, as ticks screened for arboviruses in the CAR frequently contained DUGV, but not CCHFV [10]. However, this displays only indirect evidence for cross-reactivities and needs to be verified by monospecific antisera following defined experimental infections of ruminants.

Such experimental DUGV challenge infections in sheep and bovines are described in the presented report. Moreover, we have established novel diagnostic tests for the detection of DUGV antibodies and determined antibody cross-reactivities by also running those sera in currently available commercial CCHFV diagnostic assays.

Due to their genetic and antigenic relationship, DUGV has already been used as a model for CCHFV infections. Several studies focusing on molecular biology present the in vitro characterization and comparison of both viruses, e.g., when studying apoptosis mechanisms [27] or differential activation profiles of antigen-presenting cells [28]. Ruminants are natural hosts for both viruses, but it is understudied to what extent, if at all, they develop overt or remote pathologies following those infections individually. DUGV might be a more suitable surrogate for CCHFV infections in ruminants than HAZV (CCHFV serogroup), as ruminants are not considered to be susceptible to HAZV [29].

## 2. Materials and Methods

### 2.1. Virus and Cells

The prototype strain of DUGV (IB AR 1792, GenBank accession number: KU925455, KU925456, KU925457) was kindly provided by the World Reference Center for Emerging Viruses and Arboviruses, University of Texas Medical Branch, Galveston, USA. The lyophilized stock (13th suckling mouse passage) was grown on SW13 cells, which were kindly provided by Ali Mirazimi, National Veterinary Institute, Sweden. SW13 cells were cultivated with L-15 medium (Sigma Aldrich, Darmstadt, Germany) supplemented with 5% fetal calf serum (FCS). The virus was passaged two times on SW13 cells to generate a virus stock for the animal infections. Growth kinetics revealed maximum viral titers 48 h post infection (up to 10^7,9^ TCID_50_/mL). Vero E6 cells (Collection of Cell Lines in Veterinary Medicine, Friedrich-Loeffler-Institut, Germany) were cultivated with modified Eagles medium (MEM, Collection of Cell Lines in Veterinary Medicine, Friedrich-Loeffler-Institut, Germany) supplemented with 10% FCS and utilized for indirect immunofluorescence assays.

### 2.2. Quantification

Virus titers were determined with two different methods, namely, endpoint titration (50% tissue culture infective dose, TCID_50_) and plaque assay (plaque forming units, pfu)_,_ as already described for HAZV [29]. Both assays were performed on SW13 cell monolayers. Cells were fixed with neutral buffered formalin and stained with crystal violet four and seven days post infection for the pfu and TCID_50_ titer, respectively. Comparing the different quantification methods for the same virus stock, the pfu titers were approx. 1 log scale lower than the TCID_50_ titers.

### 2.3. Quantitative Reverse Transcriptase Polymerase Chain Reaction (RT-qPCR)

To determine the load of viral genome equivalents in samples suspected to be positive for DUGV, a target region on the S segment of the viral genome was selected. The primers and probes used are listed in Table 1.

RT-qPCR was performed using the QuantiTect Probe RT-PCR Kit (Qiagen, Hilden, Germany) in a total reaction volume of 25 μL according to the manufacturer’s instructions. The reaction mix comprised 12.5 µL 2× reaction master mix, 0.25 µL RT-Mix, 2 pmol of each DUGV primer and 0.5 pmol of DUGV probe. Water was used to fill up to a volume of 20 µL and finally 5 μL of template RNA was added. As internal extraction control, MS2 bacteriophage RNA was added to the samples before RNA isolation. A specific MS2 primer/probe mix was used for detection [30]. A synthetic RNA comprising the target region of the RT-qPCR was utilized as a calibrator for quantification and as a positive control for each RT-qPCR plate. The synthetic calibrator was generated by in vitro transcription from the corresponding DNA sequence, which contains an additional T7 promotor sequence at the 5′-end for in vitro transcription.

The real time RT-qPCR was carried out using a CFX96 Real-Time PCR Detection System (Bio-Rad Laboratories, Hercules, CA, USA). Cycling conditions used were as following: 50 °C for 30 min (reverse transcription), 95 °C for 15 min (reverse transcriptase inactivation/Taq polymerase activation), followed by 45 cycles at 95 °C for 15 s (denaturation), 60 °C for 30 s (annealing) and 72 °C for 30 s (elongation). Fluorescence data were collected after each 72 °C step and analysis of the fluorescence data was conducted with CFX Manager software (Bio-Rad Laboratories, Hercules, CA, USA).

### 2.4. Recombinant Expression of DUGV N Protein

The coding region for the DUGV N protein is located on the S segment of the viral genome. For recombinant expression, the nucleic acid sequence of DUGV strain AR 44313 (GenBank accession number: AF434161) was codon optimized for expression in Escherichia coli. A synthetic plasmid comprising restriction sites for subcloning 5′ and 3′ of the coding sequence and the coding sequence itself was generated by Eurofins Genomics (Ebersberg, Germany). Besides the homologous sequences, restriction sites, NdeI and BamHI, respectively, were introduced for subcloning. Subsequently, the coding region was cloned into the prokaryotic expression vector pET19b using the restriction enzymes NdeI and BamHI. Sequence identity and correct insertion were verified by sequencing.

For recombinant expression, E.coli K12 strain BL21(DE3) was transformed with the pET19b-DUGV N plasmid. Protein expression was induced by addition of isopropyl-β-D-thiogalactopyranosid (IPTG, MP Biomedicals, Irvine, CA, USA) during the logarithmic growth phase of the bacteria. After 4 h of expression, bacteria were sedimented and protein isolation and purification steps were carried out using the N-terminal His-Tag binding to Ni-NTA Sepharose utilizing the customary expressionist protocol (Qiagen, Hilden, Germany).

### 2.5. Serology

#### 2.5.1. Development of an Indirect IgG-ELISA Based on Recombinant Antigen

Bovine protocol: The test antigen (DUGV N protein) was diluted to a final concentration of 2 µg/mL in phosphate-buffered saline (PBS) containing 0.5% BSA (albumin fraction V, Merck, Darmstadt). In total, 100 µL per well were coated on Greiner F plates for 1 h at 37 °C. For every sample, four wells were coated: two wells with the antigen and two wells with the antigen dilution buffer only. Plates were washed three times with 200 µL/well wash solution (PBS containing 0.05% Tween20). Afterwards, 200 µL of blocking buffer (IDVet, Grabels, France) were added and plates were incubated for 1 h at room temperature. Sera were diluted 1/20 in IDVet Buffer No. 3 and 100 µL/well were added. The positive control serum of an immunized calf (DUGV-IMMU-calf-1) was prediluted 1/80 in negative cattle serum, as the serum yielded extremely high antibody levels. The following incubation steps were all performed for 1 h at 37 °C. Plates were washed three times with 200 µL/well wash solution and the conjugate (Peroxidase-conjugated AffiniPure Goat Anti-Bovine IgG, Jackson ImmunoResearch, Cambridgeshire, UK) was diluted 1/10,000 in IDVet Buffer No. 11 and 100 µL were added to each well.

After rinsing the plates again, 100 µL of the substrate (1-Step^TM^ Ultra TMB-ELISA, Thermo Scientific, Braunschweig, Germany) were added to each well and incubated for approx. 10 min in the dark. The reaction was stopped with 100 µL/well 1M H_2_SO_4_ and OD values were measured at 450 nm. Corrected OD values were calculated (mean OD_450_ with antigen−mean OD_450_ without antigen) and the percentage of the corrected OD value of the sample in comparison to the positive control (immunized cattle, DUGV-IMMU-calf-1) was determined.

Ovine protocol: Only differences between the ovine and bovine protocol are mentioned. Antigen was diluted in PBS containing 1% albumin from sheep (Sigma-Aldrich, Darmstadt, Germany) and plates were coated overnight at 4° C. All washing steps were performed with PBS containing 0.1% Tween20. The secondary antibody (Peroxidase-conjugated AffiniPure Donkey Anti-Sheep IgG, Jackson ImmunoResearch, Cambridgeshire, UK) was diluted 1/5000. The positive control serum (immunized sheep, DUGV-IMMU-sheep-1) was diluted in the same manner as the test sera.

#### 2.5.2. Indirect Immunofluorescence Assay

The protocol for the indirect immunofluorescence assay (iIFA) was performed as already described for HAZV [29]. Briefly, DUGV-infected Vero E6 cells were fixed with methanol/acetone and incubated with the sera diluted 1/50 and afterwards with the corresponding secondary antibody (anti-bovine/anti-ovine).

#### 2.5.3. Plaque Reduction Neutralization Test (PRNT)

The PRNT was also performed following the protocol already published for HAZV [29]. A serum dilution was considered positive if 80% of plaques (PRNT_80_) were reduced compared to the control plate.

#### 2.5.4. Micro-Virus Neutralization Test (mVNT)

SW13 cells were seeded in 96-well plates 24 h before performing the neutralization assay. Sera were heat-inactivated for 30 min at 56 °C. A pre-dilution plate (V-bottom) was used for the serial log2 dilution of the sera in maintenance medium (L-15, 2% FCS) starting with a 1/5 ratio (final volume 50 µL). The virus stock was diluted to a final concentration of 2000 TCID_50_/mL. Fifty microliters (approx. 100 TCID_50_) were then added to the serial serum dilutions as well as to the virus control wells, which contained 50 µL cell culture medium only. One row was left uninfected and filled with 100 µL cell culture medium to serve as a negative control. Plates were then incubated for 1 h at 37 °C, 5% CO_2_ for the neutralization process.

Afterwards, the medium was removed from the prepared SW13 monolayers and 45 µL of the serum/virus mixture were transferred from the pre-dilution plate to the cells in duplicate. Plates were then incubated for 1 h at 37 °C, 5% CO_2_, before 100 µL of maintenance medium were added to each well. In parallel, back-titrations of the inoculated virus dilution were performed, reconfirming the correct dilution of the virus stock. Two- or three-day post-infection plates were evaluated with a light microscope to rule out cytotoxic effects of the sera. Seven days post infection, plates were fixed with neutral buffered formalin and stained with crystal violet. The cytopathic effect (CPE) was evaluated visually and the 50% neutralizing dose (ND_50_) was calculated according to Behrens–Kaerber. The assay was scored as valid if the cell control wells did not show CPE, the virus control wells all showed CPE, negative control sera tested negative and the back-titration revealed viral titers of 30-300 TCID_50_/50 µL. Sera were scored as positive when ND_50_ titers were at least 1/7.

#### 2.5.5. CCHFV Diagnostic Assays

DUGV antisera were run in three different CCHFV ELISA systems. (1) The multispecies double antigen ELISA (IDVet, Grabels, France) was applied according to the manufacturers’ instructions. (2) An in-house ELISA based on recombinant N protein and (3) a species-adapted protocol for the commercial human Vector-Best IgG ELISA (Novosibirsk, Russia). Moreover, an immunofluorescence assay (Euroimmun, Luebeck, Germany) was used as described before [31]. Western blot analyses were conducted according to standard procedures.

### 2.6. Immunizations

One calf (DUGV-IMMU-calf-1) and one sheep (DUGV-IMMU-sheep-1) were immunized subcutaneously with formalin-inactivated DUGV (cattle: 4.8 × 10^6^ TCID_50_, sheep: 1.42 × 10^6^ TCID_50_). Each animal received three boosts containing the inactivated virus and equivalent amounts of adjuvant (GERBU Adjuvant P, GERBU Biotechnik GmbH, Heidelberg, Germany). Each animal was injected at four different locations (breast, neck) with 2.5 mL per injection site.

### 2.7. Animal Trials

Animal trials were conducted under BSL 3 conditions. Four sheep (German mutton, 2 female/2 male, age 5–7 months, obtained from Friedrich-Loeffler-Institut, Mariensee, internal animal code: G5, G6, H5, H6) and four calves (Holstein Friesian and German Black Pied cattle, 2 female/2 male, age 4–6 months, obtained from commercial supplier RinderAllianz GmbH and from Friedrich-Loeffler-Institut, Mariensee, internal animal code: J5, J6, K5, K6) were inoculated subcutaneously in the lateral chest wall with 10^6^ TCID_50_ DUGV (injected volume 3 mL). As mock controls, one sheep (internal code: I2) and one calf (internal code: L2) were kept separate and inoculated with cell culture medium only.

Rectal body temperature was measured daily and clinical scores (considering general condition, behavior, activity, breathing rate, food uptake and injuries) were determined. Blood (whole blood and serum) and swab (nasal and rectal) samples were collected prior to infection and at different time points post infection (1st week: every day, alternating half of the animals, 2nd–4th week: every 2nd day, sampling of half of the animals).

Blood was centrifuged, serum separated and swab samples were shaken for 30 min in 1 mL of L-15 medium. These samples as well as EDTA blood were stored at −70 °C prior to RNA isolation.

Animals were euthanized intravenously with pentobarbital (Release^®^, WDT, Garbsen, Germany) 27 or 28 dpi and dissections were conducted by a specialized pathologist. A diverse panel of tissue samples was collected (injection site (lateral chest wall), Ln. cervicalis cran., heart muscle, lung, small intestine, large intestine, rectum, liver, kidney, spleen, Lnn. iliaci med., Lnn. retropharyngeales med., tonsils and brain).

Tissue samples were homogenized in 1 mL of L-15 medium using a tissue homogenizer (geneye UPHO, nbs scientific, Weinheim, Germany). Total RNA from the organ homogenates and serum and whole blood samples was extracted using the King Fisher 96 Flex purification system (Thermo Scientific, Braunschweig, Germany) in combination with the NucleoMag Vet Kit (Macherey-Nagel, Düren, Germany) according to the manufacturers’ instructions. As an internal extraction control, an MS2 bacteriophage was added prior to RNA extraction to each sample in order to avoid false-negative results in the following DUGV RT-qPCR.

## 3. Results

### 3.1. DUGV Challenge Study: Clinical Signs, Gross Pathology and Viral Genome Detection

Four sheep and four calves were inoculated subcutaneously with 10^6^ TCID_50_ DUGV. Following all practical procedures, virus concentrations of the injected fluids were reconfirmed by back-titrations (standard titrations reconfirming the actual virus titer). Animals were kept for 28 days and clinical scores were determined daily and blood as well as swab samples were collected regularly.

**Figure 1:**
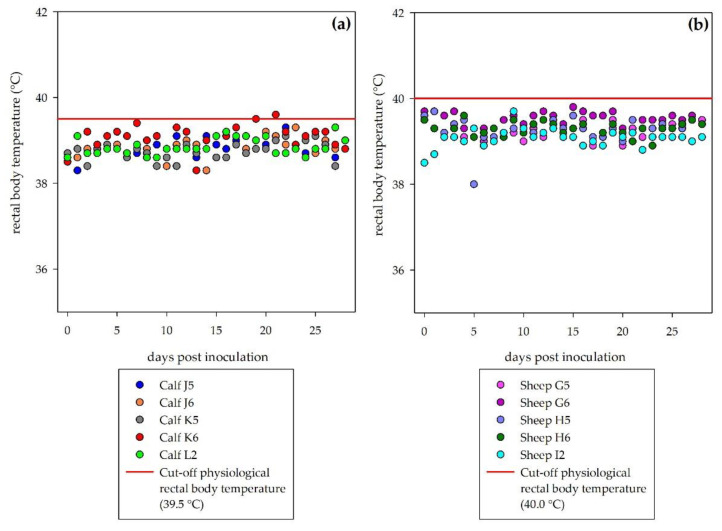
Rectal body temperature during the observation period: No fever was detected in inoculated cattle (**a**) or sheep (**b**). Physiological rectal body temperatures can rise up to 39.5 °C in calves and up to 40.0 °C in sheep (illustrated by the red line in each graph), short-term higher temperatures may be animal handling/excitement artefacts.

Neither sheep nor calves developed clinical signs following DUGV inoculation. No rise of the rectal body temperatures was recorded (Figure 1). During the necropsies, no gross pathological lesions related to viral disease were observed.

RT-qPCR analysis did not lead to positive results in any of the collected blood, swab and tissue samples. However, the successful RNA extraction was proven, as MS2 extraction controls were detected in all samples.

### 3.2. Establishment of Indirect DUGV-ELISAs

#### 3.2.1. Cattle

**Figure 2:**
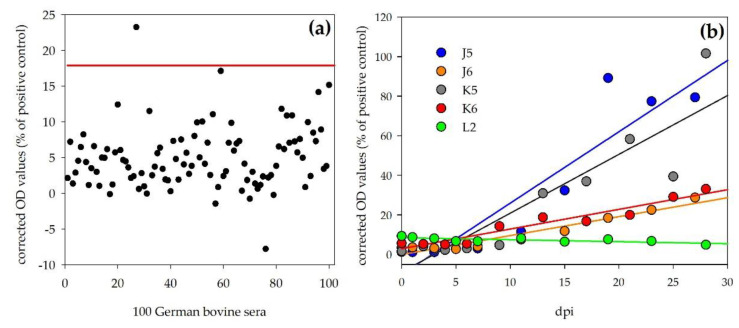
Bovine DUGV ELISA: (**a**) Results of 100 negative German reference sera for the determination of the negative cut-off. Diagnostic specificity for this ELISA was 99%. (**b**) Significant increase in OD values (shown by linear regression of ELISA values; calculations done by Sigmaplot) during the observation period for the four inoculated calves (J5, J6, K5, K6), whereas the OD values for the control animal (L2) stayed at the same level during the whole experiment.

Sera of the four inoculated calves (J5, J6, K5, K6) and of the mock control (L2) were tested with the novel indirect DUGV ELISA. Corrected OD values (percentage of positive control: DUGV-IMMU-calf-1) showed a steady increase during the observation period for all four inoculated calves (Figure 2b). However, two different immune responses were observed: J5 and K5 developed higher antibody levels (28 dpi, J5: 79.4%, K5: 101.6%) than J6 (28 dpi, 28.7%) and K6 (28 dpi, 33.1%).

For the determination of the negative cut-off value, 100 sera from German cattle were tested with this ELISA and the negative cut-off was calculated as follows: mean + 3 × standard deviation = 17.9%. Using this cut-off, the diagnostic specificity was 99% (Figure 2a). Time points (days) post inoculation of the individual animals are listed in Table 2 after which this cut-off value was passed in comparison to the results obtained by iIFA and mVNT. The OD values of the control animal (L2) stayed at the same level during the experiment and were below the determined negative cut-off at any time post inoculation (e.g., 28 dpi, 4.9%).

#### 3.2.2. Sheep

One hundred sera from German sheep were tested with the indirect DUGV ELISA and obtained results were used for the determination of the negative cut-off (mean + 3 × standard deviation = 16.2%). With this cut-off value, diagnostic specificity was 98%. The immunized sheep (DUGV-IMMU-sheep-1) reacted with the DUGV antigen and served as a positive control and reference serum for the calculation of the corrected OD values.

**Figure 3:**
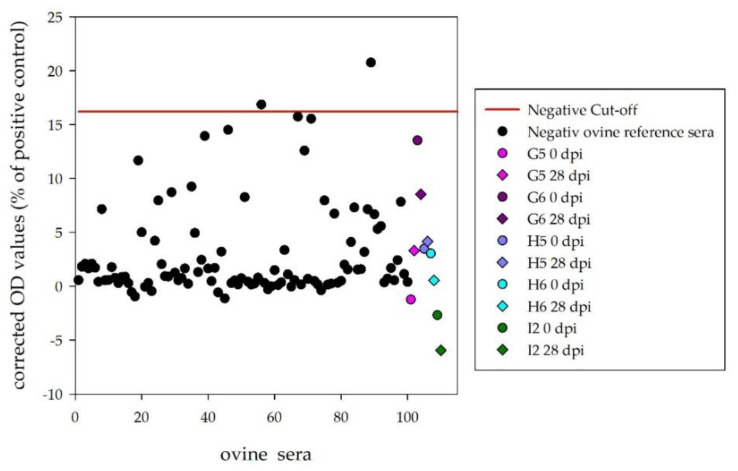
Ovine DUGV ELISA: 100 negative German sheep sera were used to determine the ELISA cut-off. OD values of the four inoculated sheep (G5, G6, H5, H6) and the control animal (I2) were below the determined cut-off prior to and post inoculation (28 dpi).

Sera of the four inoculated sheep and the mock control were tested with this ELISA. Corrected OD values (28 dpi) were below the determined cut-off for G5 (3.3%), G6 (8.5%), H5 (4.2%), H6 (0.6%) and I2 (−5.9%) (Figure 3).

### 3.3. iIFA

#### Cattle

**Figure 4:**
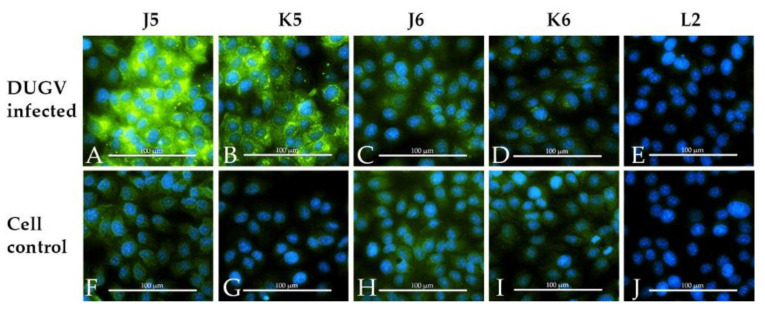
Indirect immunofluorescence assay with Vero E6 cells infected with DUGV (**A**–**E**) and non-infected control wells (**F**–**J**). Calf J5 (**A/F**), K5 (**B/G**), J6 (**C/H**) and K6 (**D/I**) show a specific staining of DUGV-infected cells, whereas the serum of the mock control L2 (**E/J**) tested negative.

The sera of the inoculated calves led to a specific fluorescence signal on DUGV-infected cells compared to the non-infected control wells (Figure 4). However, the signal was quite strong for J5 and K5, but weak for J6 and K6 (only staining of small marked areas in the cytoplasm). The days post infection when the sera were scored as positive for the first time are presented in Table 2.

The sera of the inoculated sheep only led to a very weak fluorescence signal, which was solely detected in direct comparison with the non-infected control wells (comparable to the results depicted for calf J6 and K6).

### 3.4. PRNT

All inoculated animals developed neutralizing antibodies demonstrable by the PRNT (Table 3). Sera before inoculation as well as the sera of the control animals (I2, L2) did not show a significant reduction of plaques.

**Figure 5:**
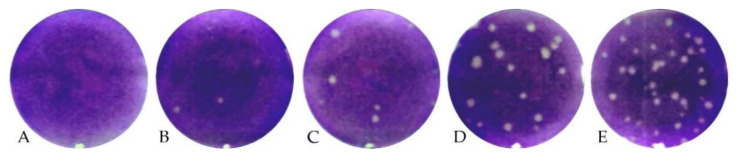
Plaque reduction neutralization test (PRNT) of calf J6 (28 dpi). Serial 1/2 dilutions of the serum from 1/8 (**A**) to 1/128 (**E**) show a steady increase in plaques. Eighty percent of plaques were reduced up to 1/32 (**C**).

As already shown for the ELISA and iIFA, two patterns were observed within the tested cattle sera: J5 and K5 revealed higher levels of neutralizing antibodies (PRNT_80_: 1/256) than J6 and K6 (PRNT_80_: 1/32). An example for the PRNT of calf J6 is presented (Figure 5). The four sheep reacted quite homogenously, with PRNT_80_ titers of 1/16 (G6 and H5), 1/32 (G5) and 1/64 (H6).

The immunized ruminants did also develop neutralizing antibodies, with PRNT_80_ titers of 1/512 for the immunized sheep and 1/256 for the calf (Table 3).

### 3.5. mVNT

**Figure 6:**
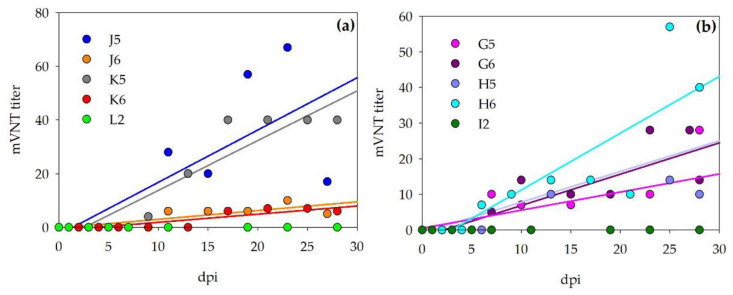
Micro-virus neutralization test (mVNT) results for inoculated cattle (**a**) and sheep (**b**). mVNT titers during the observation period are visualized and show a steady increase in neutralizing antibodies for the inoculated ruminants (visualized by linear regression of mVNT titers; calculations done by Sigmaplot), whereas the control animals did not develop neutralizing antibodies.

Neutralizing antibodies were also detected by mVNT for all of the infected animals. Titers beginning with 1/7 were scored positive. Sera before inoculation as well as sera of the control animals did not test positive. Titers are visualized for the calves and sheep, respectively, in Figure 6.

Table 2 presents an overview of after which time points (dpi) each assay (ELISA, iIFA or mVNT) indicated a positive test result for the inoculated cattle. For the sheep sera, only mVNT results are presented, as the sera did not lead to positive results in the ELISA and iIFA signals were too weak for a precise determination of the first positive day.

### 3.6. Reactivity of DUGV Antisera in CCHFV Diagnostic Assays

Results concerning the evaluation of cross-reactivities between DUGV and CCHFV are summarized in Table 4.

**Figure 7:**
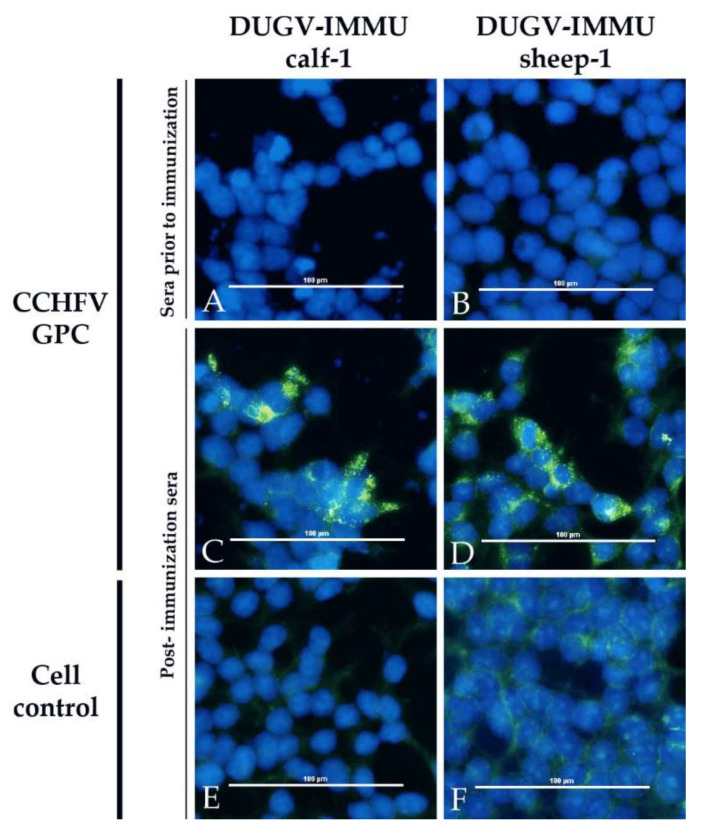
CCHFV immunofluorescence assay with DUGV hyperimmune sera: Cells expressing CCHFV GPC (**A**–**D**) and non-transfected controls (**E**+**F**) are depicted. The post-immunization serum of the immunized calf (DUGV-IMMU-calf-1) was diluted 1/300 (**A**+**C**+**E**) and the serum of the immunized sheep (DUGV-IMMU-sheep-1) was diluted 1/20 (**B**+**D**+**F**). Sera prior to infection (**A**+**B**) did not lead to a positive signal on cells transfected with CCHFV GPC, but post-immunization sera were clearly positive (**C**+**D**).

**Figure 8:**
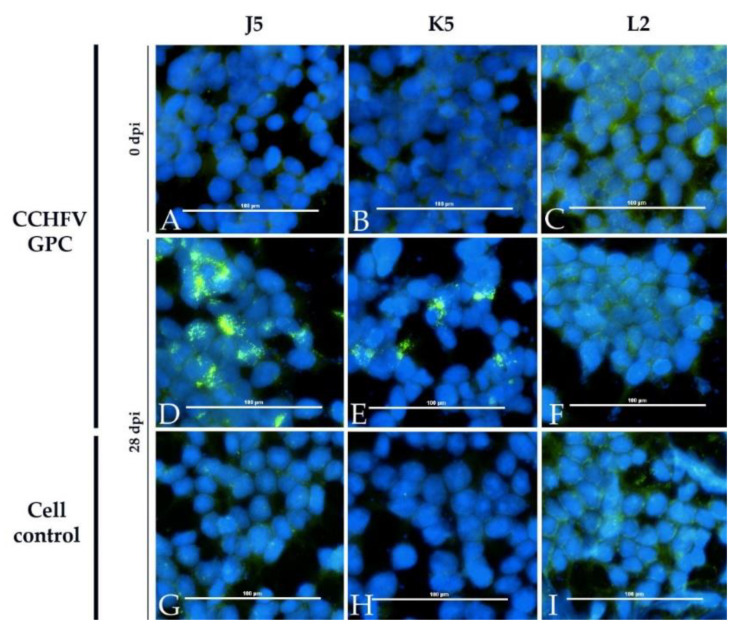
CCHFV immunofluorescence assay with DUGV cattle antisera: Cells expressing CCHFV GPC (**A**–**F**) and non-transfected controls (**G**–**I**) are depicted. Calf J5 (**A**+**D**+**G**) and K5 (**B**+**E**+**H**) show a specific staining of CCHFV GPC-transfected cells, whereas the serum of the control calf L2 (**C**+**F**+**I**) did not test positive. Sera prior to infection (**A**–**C**) did not lead to a positive signal on cells transfected with CCHFV GPC.

In the CCHFV iIFA, sera of the immunized animals (Figure 7) as well as sera of the two calves, which developed higher levels of DUGV antibodies, tested positive (Figure 8).

**Figure 9:**
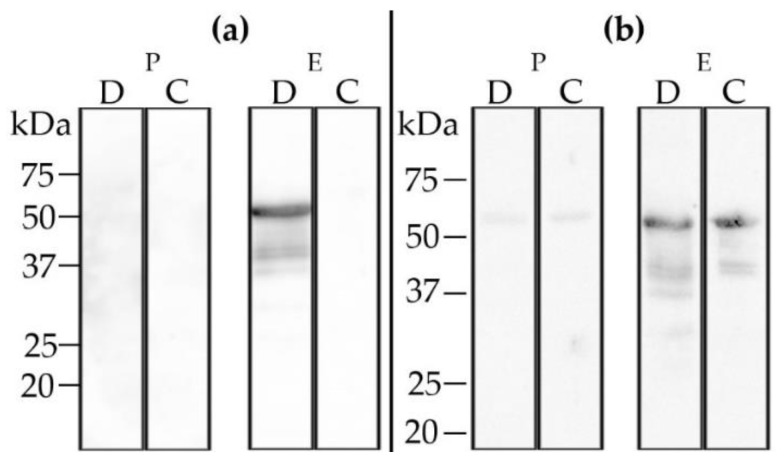
Western blot analysis with recombinant DUGV (D) and CCHFV (C) N protein: Post-immunization sera (E) of the immunized calf (DUGV-IMMU-calf-1) (**a**) and the immunized sheep (DUGV-IMMU-sheep-1) (**b**) strongly react with the DUGV N protein. The sheep serum cross-reacts with the CCHFV N protein, whereas the calf serum reacts specifically with the DUGV N protein only. Sera prior to immunization (P) did not test positive.

Western blot analysis employing the post-immunization serum of the immunized sheep (DUGV-IMMU-sheep-1) revealed weak cross-reactions to the CCHFV N protein (Figure 9).

## 4. Discussion

According to this study, sheep and cattle show low susceptibilities to experimental DUGV challenges, as the animals did not develop clinical disease or viremia. These observations are in line with previous studies and field observations. However, despite this clinical inconspicuousness in the challenged sheep and bovines, all animals produced substantial DUGV-specific antibody levels.

RT-qPCR analysis of blood (whole blood, EDTA), swab (nasal, rectal) and tissue samples did not reveal any positive results. As DUGV is a tick-borne arbovirus, it was not expected that the animals would shed virus via the respiratory or alimentary tract. Therefore, the negative results for the swab samples were not surprising. All the examined tissue samples (15 organs/animal) were also negative, although virus isolations from cattle liver specimens in Nigeria have been reported [32]. As the necropsies in this experiment were performed four weeks post inoculation, local virus infections in organs may have occurred in the early phase post infection, but animals eventually cleared the virus before euthanasia.

The negative RT-qPCR results for the blood samples are more difficult to explain. Actually, the successful isolation of DUGV out of cattle blood (via suckling mouse inoculation) was already described by different authors, implying that cattle indeed develop transient viremia following DUGV infection [7,32,33]. Moreover, DUGV was isolated from the blood of an experimentally infected calf [34]. This stands in conflict with our findings, particularly as the same virus strain and inoculation route were employed in both studies and RT-qPCRs are more sensitive than virus isolations. Differences in the applied virus titer cannot directly be compared (TCID_50_ vs. suckling mouse doses), but might offer a plausible explanation, as well as differences in the age and breed of the infected calves. In a former CCHFV infection study, the relevance of animal age for the disease outcome is exemplified. Two- and six-month-old calves have been infected with CCHFV, but viremia was only detected in the two-month-old calves [35]. Another reason for the different outcome might be the source of the inoculated virus stock. Whereas in the former study, suckling mouse brain homogenates were utilized, we additionally passaged the virus three times in cell culture before these infection trials. This might have led to a cell culture adaptation, which may have had an impact on the virulence of the isolate. The experimental inoculation of sheep with DUGV was also described in a study from the late 1980s. In this older study, animals did not develop viremia, but rather DUGV-specific antibodies, which is consistent with our results [36].

As the natural transmission of arboviruses actually requires a viremic host species, it can be argued that ruminants do not play an essential role in the infection cycle, as we were unable to detect viremia in this experiment. However, field infections may differ from experimental inoculations and actually lead to a viremic state in the infected ruminants. Differences between the artificial inoculation (subcutaneous injection) applied within the animal experiments and natural infections via tick bite may affect the outcomes. So-called saliva assisted transmission (SAT) might influence virus infectivity, as the tick saliva is thought to contain molecules modulating the locale immune response [37]. Further investigations must show whether ruminants develop viremia (detectable by RT-qPCR) after natural infections. However, even if ruminants do not develop a prolonged and high-level viremia, they may contribute to the natural transmission cycle of DUGV, when non-viremic transmission (NVT) is considered. Multiple ticks attached to a single host can transmit the virus to co-feeding ticks—without the requirement of a viremic host [38]. For CCHFV, this phenomenon was investigated in one study involving non-viremic guinea pigs and *Hyalomma* ticks [39]. Nevertheless, it is not yet clear whether NVT contributes significantly to the maintenance of *Orthonairoviruses* in general.

As most of the DUGV-positive ticks have been collected from ruminants and as investigations in Africa have revealed seroconversion and viremia in ruminants, we still assume that these vertebrates play a role in the natural infection cycle; hence, the serological monitoring of ruminants will be useful for the determination of the current DUGV distribution.

All four inoculated calves developed DUGV-specific antibodies, as shown by the serological assays (ELISA, iIFA, mVNT and PRNT) newly developed in this project. Diagnostic specificities and sensitivities of the assays need to be further evaluated using larger serum panels. Likewise, all four challenged sheep developed DUGV-specific neutralizing antibodies (see mVNT and PRNT), although negative results were obtained with the N protein-based DUGV ELISA. The immunogenicity and correct epitope presentation of recombinant N protein were verified as the sera of the DUGV-challenged calves specifically reacted with the same antigen. Moreover, the functionality of the sheep-specific ELISA protocol was shown as the serum of the immunized sheep clearly tested positive. We therefore conclude that the infected sheep did not develop detectable DUGV N-specific antibodies, although this protein is usually the dominant antigen of *Orthonairoviruses* [40]. For Schmallenberg orthobunyavirus (SBV)—another important member of the order *Bunyavirales*—similar results were obtained when pigs were experimentally inoculated with SBV. The animals did not develop detectable levels of N protein-specific antibodies, but rather low titers of SBV-neutralizing antibodies—indicating that the neutralization assays might be more sensitive than N protein-based ELISA systems [41]. Furthermore, the DUGV-challenged sheep may have produced viral glycoprotein-specific antibodies, which also commonly have neutralizing features [42]. Further studies are therefore needed to elucidate this immune response to these DUGV antigens.

Concerning the evaluation of cross-reactivities between DUGV and CCHFV, the hyperimmune sera (ovine, bovine) as well as the sera of the infected animals were employed. Three different CCHFV ELISA systems were utilized with the DUGV antisera. The double antigen ELISA could clearly discriminate between these antibodies, as all of the DUGV antisera tested negative. The CCHFV in-house and Vector-Best ELISAs could only be assessed with the cattle sera. The infected calves tested negative in both tests, whereas the serum of the DUGV-immunized calf (DUGV-IMMU-calf-1) reacted positively in the in-house CCHFV ELISA. The best discrimination was achieved by the double-antigen ELISA and the Vector-Best ELISAs.

Strong non-specific antisera binding (most likely to BSA) of all challenged and immunized sheep was seen in the in-house and Vector-Best ELISAs, whereas neither a non-specific nor CCHFV cross-reaction was observed in the IDVet double antigen ELISA.

Cross-reactions were also observed in the iIFA (Euroimmun) on cells expressing CCHFV GPC (glycoprotein precursor): bovine and ovine hyperimmune sera (DUGV-IMMU-calf-1, DUGV-IMMU-sheep-1) as well as the two stronger bovine DUGV antisera (J5 and K5) gave clear CCHFV antigen signals. Keeping in mind that DUGV and CCHFV actually do not belong to the same serogroup, these findings raise concerns about the specificity of the iIFA.

## 5. Conclusions

According to this study, ruminants are susceptible to experimental DUGV infection, with cattle being more susceptible than sheep. Several novel diagnostic assays were developed. Their diagnostic sensitivity and specificity now need to be determined on DUGV-infected ticks (RT-qPCR) and on field sera (RT-qPCR and serological assays) with unknown infection histories. When CCHFV monitoring studies are performed in regions where DUGV is also prevalent, special emphasis must be put on the discrimination of these antibodies (e.g., performing additional ELISAs as confirmative tests for positive iIFA results).

## Figures and Tables

**Table 1:** Sequences of primers and probes used for the detection of Dugbe orthonairovirus (DUGV) genome.

**Primer/Probe** **DUGV** **S Segment**	**Sequence 5′→3′**	**Localization According to Reference Sequence (GenBank Accession** **Number: AF434164)**
DUGS581F	GGAATGTCCTGCTGAACGGAGACGG	581–605
DUGS776R	AGCTCAGCAACCTTCACCATGGCA	776–753
DUGV642P	FAM-GCATGTCTCTTGGGGCCGTGAGTTGG-TAMRA	642–667
**Primer/Probe Internal** **Extraction** **Control**	**Sequence 5′→3′**	**Localization According to Reference Sequence (GenBank Accession** **Number: MK213795)**
MS2F	CTCTGAGAGCGGCTCTATTGGT	2233–2254
MS2R	GTTCCCTACAACG AGCCTAAATTC	2333–2310
MS2probe	HEX-TCAGACACGCGGTCCGCTATAACGA-BHQ1	2278–2302

**Table 2:** Comparison of first detection of DUGV antibodies by individual assays.

Challenge Animals		DUGV Diagnostic Assay		
		ELISA (>Cut-Off)	iIFA (≥1/50)	mVNT (≥1/7)
**Calves**	J5	15 dpi	15 dpi	11 dpi
	J6	19 dpi	23 dpi	23 dpi
	K5	13 dpi	9 dpi	13 dpi
	K6	13 dpi	13 dpi	21 dpi
	L2	Negative	Negative	Negative
**Sheep**	G5	Negative	ND	7 dpi
	G6	Negative	ND	11 dpi
	H5	Negative	ND	9 dpi
	H6	Negative	ND	9 dpi
	I2	Negative	Negative	Negative

ND: not determined, iIFA: indirect immunofluorescence assay, mVNT: micro-virus neutralization test.

**Table 3:** PRNT_80_ titers of inoculated and immunized animals.

Animal		PRNT_80_ Titer 28 dpi
**Inoculated**	J5	1/256
**Calves**	J6	1/32
	K5	1/256
	K6	1/32
**Mock Control**	L2	<1/8
**Inoculated**	G5	1/32
**Sheep**	G6	1/16
	H5	1/16
	H6	1/64
**Mock Control**	I2	<1/8
**DUGV-IMMU-sheep-1**		1/512 *
**DUGV-IMMU-calf-1**		1/256 *

* Post immunization sera after 3 boosts.

**Table 4:** Results obtained with Crimean-Congo hemorrhagic fever orthonairovirus (CCHFV) assays and DUGV antisera.

Diagnostic Assay Incl. Antigen		ELISA			iIFA	Western Blot
		Vector-Best	IDVet Double Antigen	In-House	Euroimmun	In-House
		Inactivated whole virus, clade IV	Recombinant N protein, clade III	Recombinant N protein, clade V	Transfected cells with GPC and N protein, clade III	Recombinant N protein, clade V
**Serum**	Immunized calf	Negative	Negative	Positive	Positive	Negative
	Immunized sheep	*	Negative	*	Positive	Positive
	Infected calves	Negative	Negative	Negative	J5 + K5 positive	ND
	Infected sheep	*	Negative	*	Negative	ND

* The post-immunization/infection sera led to strong non-specific reactions. GPC: glycoprotein precursor

## Data Availability

The data presented in this study are available within this manuscript, Hartlaub et al., *Viruses*.
